# Hashimoto's encephalopathy with cerebellar ataxia as the main symptom: A case report and literature review

**DOI:** 10.3389/fneur.2022.970141

**Published:** 2022-08-23

**Authors:** Chunxiao Wei, Yanxin Shen, Weijie Zhai, Tianling Shang, Zicheng Wang, Yongchun Wang, Mingxi Li, Yang Zhao, Li Sun

**Affiliations:** Department of Neurology and Neuroscience Center, The First Hospital of Jilin University, Jilin University, Changchun, China

**Keywords:** Hashimoto's encephalopathy, Hashimoto's autoimmune thyroiditis, ataxia, antithyroid antibodies, autoimmune thyroiditis

## Abstract

Hashimoto's encephalopathy (HE), also known as steroid responsive encephalopathy associated with autoimmune thyroiditis (SREAT), has a variety of clinical manifestations, with various neuropsychiatric characteristics, including tremors, transient aphasia, seizures, altered consciousness, myoclonus, cognitive impairment, and psychiatric manifestations. The hallmark presenting feature is a non-specific encephalopathy characterized by alteration of mental status and consciousness ranging from confusion to coma and impaired cognitive function, while those with cerebellar ataxia as the main manifestation is rare. We reported a case of Hashimoto's encephalopathy with cerebellar ataxia as the main manifestation, elevated anti-thyroid antibodies (anti-TPO/TG), and normal thyroid function. The symptoms of cerebellar ataxia improved after steroid treatment. Meanwhile, we reviewed the clinical features of 20 representative cases of HE with cerebellar ataxia as the core symptoms. In conclusion, based on our case findings and literature review, the diagnosis of HE should be suspected in cases of encephalopathy without an obvious cause, to quickly start an effective treatment.

## Introduction

Hashimoto's encephalopathy (HE) is an autoimmune encephalopathy related to thyroid antibodies, which was first reported by Brain et al. in 1966 (1). It is believed that HE is related to the autoimmune reaction secondary to Hashimoto's thyroiditis, and the immune inflammatory reaction during the pathogenic process may lead to focal or diffuse brain damage in the brain, leading to clinical symptoms such as unconsciousness or focal neurological loss including cognitive impairment, tremor, altered consciousness, transient aphasia, seizures, myoclonus, gait disorder/ataxia (2). To date, clinical reports of cerebellar ataxia as the main symptom of HE are rare. Meanwhile, there are no clear diagnostic criteria for HE, so it is easy to be ignored or misdiagnosed in clinical practice.

Here we report a rare case of HE with acute cerebellar ataxia as the main manifestation, elevated anti-thyroid antibodies (anti-TPO/TG), and normal thyroid function. In addition, we summarized its clinical features, diagnosis, and treatment in conjunction with the literature to enhance the understanding of HE in clinical practice, expecting to reduce the missed diagnosis rate of the disease. Furthermore, we retrieved 20 representative patients diagnosed as HE with cerebellar ataxia as the main symptom from PubMed and reviewed their etiology, clinical manifestations, auxiliary examination, diagnosis, prognosis, and treatment.

## Case presentation

**Figure 1:**
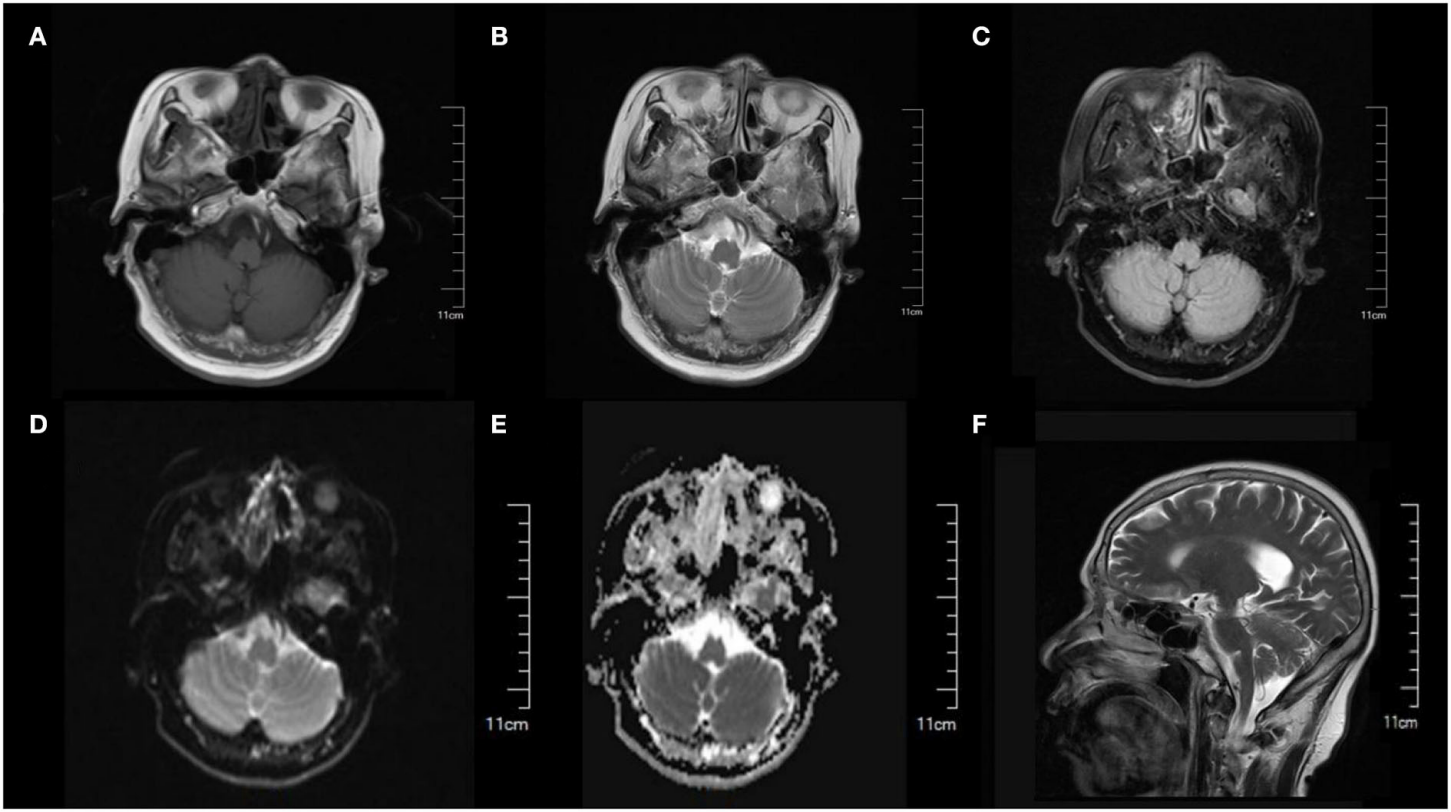
Cranial MRI showed normal brain structure and no obvious abnormal signal was found. **(A)** T1-weighted image. **(B)** T2-weighted image. **(C)** FLAIR image. **(D)** DWI image. **(E)** ADC image. **(F)** Sagittal section image.

A 64-year-old female with a complaint of dizziness and gait instability for 1 month was admitted to our hospital. The patient had a history of upper respiratory tract infection 2 days before the onset of these symptoms, which was characterized by nasal congestion and throat dryness, with no significant fever, cough, or expectoration. 2 days later, dizziness and gait instability with visual rotation, nausea, and intermittent vomiting occurred and progressively aggravated within 1 month. There was no headache, limb numbness, unconsciousness, or convulsive episodes during the course of the disease. No previous specific disease history, no drug exposure or alcohol consumption history, and no vaccination history in the last 1 year. On admission, physical examination revealed normal blood pressure of 132/83 mmHg, ataxic dysarthria, gross rotational nystagmus during horizontal and vertical eye movements, normal muscle strength of limbs, no eyelid ptosis, eye movement disorders or pupillary changes, no diminished or absent tendon reflexes, unstable bilateral finger-nose test and heel-knee-shin test, and truncal ataxia. We performed the first assessment of the severity of the patient's cerebellar ataxia using the Scale for the Assessment and Rating of Ataxia (SARA), with a score of 37. Serological examinations including blood routine, C-reactive protein, serum vitamin B1, vitamin B12, folic acid, human immunodeficiency virus (HIV) antibody, as well as other autoimmunity markers including antinuclear antibody (ANA), anti-neutrophil cytoplasmic antibodies (ANCA), and rheumatoid factors were all unremarkable. Imaging examination including cranial magnetic resonance imaging (MRI) (Figure 1) and lung computed tomography (CT) was normal. Transcranial doppler (TCD) revealed there were no cerebrovascular stenosis, atherosclerosis, or other abnormalities. Breast and abdominal ultrasounds were normal. Lumbar puncture revealed clear cerebrospinal fluid (CSF) with an opening pressure of 100 mmH_2_O. CSF biochemical tests showed slightly increased protein levels (0.47 g/L, reference range (RR): 0.15–0.45 g/L), positive Pan's reaction, while glucose and chlorine levels were normal. CSF cytology showed normal white blood cells (4.00 × 10^6^/L, reference range (RR): 0.00–8.00 × 10^6^ g/L) and visible lymphocytes, no cancer cells were seen. CSF results for infections (rubella virus, cytomegalovirus, and herpes simplex virus antibodies, general bacteria, fungi, and tuberculosis smears) were negative. Antibody analysis of autoimmune encephalitis and paraneoplastic syndrome including anti-NMDAR, anti-AMPA1, anti-AMPA2, anti-LGI1, anti-CASPR2, anti-GABA, anti-Hu, anti-Yo, anti-Ri, anti-MA2, anti-CV2, anti-Amphiphysin, anti-Tr (DNER), anti-Zic4, anti-SOX1, and anti-GAD65, were all negative in both serum and CSF.

**Figure 2:**
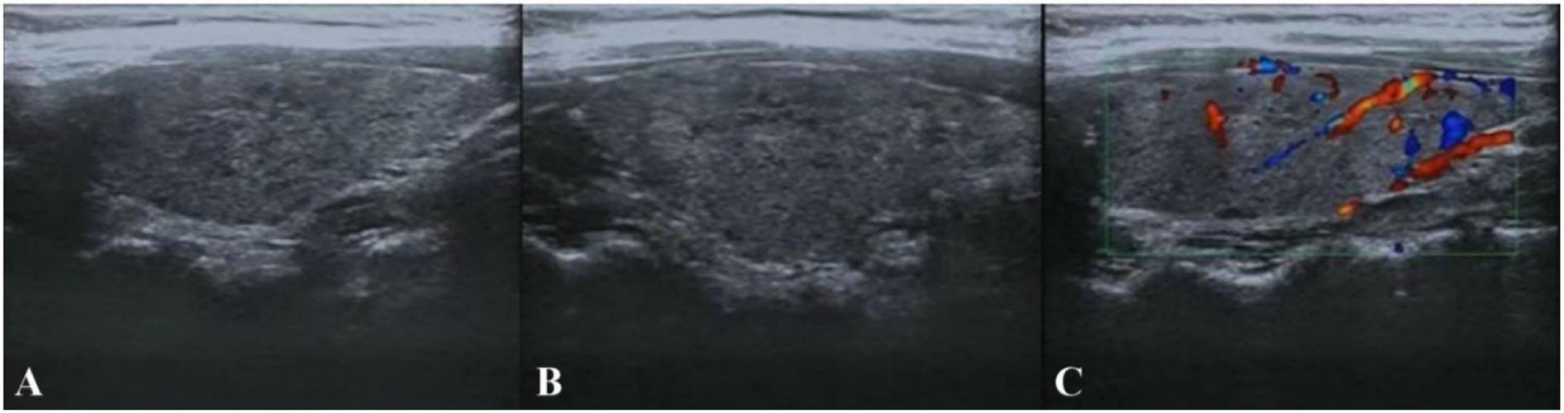
Thyroid color ultrasound. **(A,B)** The size and morphology of the left and right lobes of the thyroid gland were normal, the thickness of the isthmus was normal, the border was clear, the envelope was intact, the internal echogenicity was diffuse rough and heterogeneous, lamellar hypoechogenicity was seen. **(C)** No abnormal flow signals were seen.

Based on the above clinical data, the patient was first considered to have acute cerebellitis that might have been caused by a certain virus infection. The clinical conditions usually improved after 3–5 days of antiviral therapy in acute cerebellitis caused by viruses (3, 4). Another patient with acute cerebellitis caused by the virus received antiviral and low-dose dexamethasone (10 mg four times daily) treatment for a total of 4 days, with significant improvement of symptoms within 7 days (5). We referred to previous experience and gave antiviral and medium-dose steroid treatment (methylprednisolone 80 mg/day) for 7 days while cerebellar symptoms did not improve. Considering that the patient's symptoms lasted for more than 1 month with poor antiviral and medium-dose steroid treatment effects, whether there were other factors causing cerebellar ataxia, thyroid color ultrasound, and serum thyroid function were examined. Thyroid color ultrasound (Figure 2) showed that the internal echogenicity was diffuse, rough, and heterogeneous, and lamellar hypoechogenicity was seen, supporting the diagnosis of Hashimoto's thyroiditis. Serum thyroid function showed slightly decreased thyroid stimulating hormone (TSH, 0.3006 uIU/ml, RR: 0.35–4.94 uIU/ml), normal free triiodothyronine (FT3, 4.21 pmol/L, RR: 4.11–6.47 pmol/L), normal free thyroxine (FT4, 13.54 pmol/L, RR: 9.01–19.05 pmol/L), while significantly elevated anti-thyroglobulin autoantibodies (Tg-Ab, 87.72 IU/ml, RR: 0–4.11 IU/ml) and anti-thyroperoxidase autoantibodies (TPO-Ab, 13.18 IU/ml, RR: 0–5.61 IU/ml).

According to the main symptoms of cerebral ataxia, almost normal neuroimaging and CSF tests, by excluding other toxic, metabolic, and infectious causes of encephalopathy, the patient was diagnosed with HE. The patient was given the steroid methylprednisolone (1 g/d) for 3 days and reduced to oral prednisolone 60 mg/d, and then gradually reduced within 2 months to a maintenance dosage of 20 mg/day for 1 month. The patient's symptoms began to show improvement on day 3 of high-dose steroid therapy. 1 month later, her RASA score was 3 and she was discharged from the hospital with a marked improvement in ataxia, showing only slight dysarthria, and less obvious limb ataxia. After follow-up, the patient's symptoms were completely relieved within 3 months. In addition, after being discharged from our hospital, the patient completed a whole-body imaging Positron Emission Tomography Computed Tomography (PET-CT) examination at another hospital with no suspicious positive findings within 1 month.

## Discussion

Common etiologies of acute cerebellar ataxia include stroke, vitamin deficiency, infectious, toxic, immune-mediated, paraneoplastic, structural, and metabolic diseases (6, 7). In the present case we reported, diseases such as alcoholic cerebellar degeneration, intoxication (drugs and metals), vitamin deficiency (vitamin B1 and B12), cerebellar stroke, tumors, benign paroxysmal torticollis, and migraine with brainstem aura and other vestibular disorders were excluded. Finally, acute cerebellitis due to viral infection was first considered. Then, the patient was given antiviral and medium-dose steroid treatment for 7 days with poor results. Subsequent abnormity of anti-thyroid antibodies and thyroid color ultrasound and together with significant effects of high-dose steroid therapy led to the final diagnosis of HE.

HE is also known as steroid-responsive encephalopathy associated with autoimmune thyroiditis (SREAT) (8) is a rare disease and it is difficult to estimate its incidence and prevalence. One prospective study summarized 26 cases of unexplained encephalopathy with detectable antithyroid antibodies and estimated a prevalence of 2.1/100,000 subjects (9). Patients are predominantly female and the proportion of males and females is 1:4.1 (10, 11). Usually, disease occurs in their 5–6th decades of life (12, 13). The prevalence in pediatric populations is relatively lower than that in adults (14).

### Pathogenesis

It is commonly believed that the clinical manifestations of HE are associated with high levels of thyroid peroxidase (TPO) and thyroglobulin (TG) antibodies, but the exact underlying pathogenesis of HE is still unknown. Primarily, pathogenesis based on the presence of cerebral vasculitis was supported by the presence of perivascular lymphocytic inflammation in the brain tissue samples of some HE patients (15, 16). However, more scholars considered HE as an autoimmune disease because it is closely associated with autoantibodies that interact with shared thyroid antigens and respond well to steroid therapy (12, 17). In addition, serum anti-TPO antibodies in the CSF have also been measured in patients diagnosed with HE along with autoimmune reactions of these antibodies with the cerebral vascular and brain astrocytes that result in either vasculitis or damage to the brain cells (18, 19). Furthermore, it does not appear to be directly related to hypothyroidism or hyperthyroidism, and plasma antithyroid antibody titers do not correlate notably with the severity of HE (19–21). In addition, antithyroid antibody titers remain discoverable after treatment.

A range of autoimmune diseases has been associated with Hashimoto's thyroiditis, including rheumatoid arthritis, systemic lupus erythrematosus, ulcerative colitis, pernicious anemia, myasthenia gravis, multiple sclerosis, and thyroid ophthalmopathy (22). Simultaneously, in the cases we summarized, one patient (23) had ulcerative colitis before the onset of the disease, and five patients (5/20, 25%) had a previous history of Hashimoto's thyroiditis, which seems to prove that autoimmunity plays an irreplaceable role in the pathogenesis of HE.

### Clinical presentations

The clinical manifestations of HE are diverse and the course is generally recurrent and remitting. The most common features of HE are subacute episodes of altered level of consciousness, stroke-like episodes, and myoclonus. Kothbauer-Magreiter et al. (17) have described two distinct presentations of HE, where the clinical overlap is common. The first is a vasculitic type with repetitive stroke-like episodes, such as hemiparesis, aphasia, and ataxia with only mild cognitive impairment. The second is an inert progressive type with an insidious onset of dementia, seizures, hallucinations, psychotic episodes, or altered consciousness. Seizures, comas, tremors, and myoclonus can occur in both types (17, 24). The hallmark presenting feature is a non-specific encephalopathy characterized by altered mental status and consciousness, ranging from confusion to coma and impaired cognitive function (25). Seizures, including both partial and generalized seizures (11, 26), and myoclonus have been reported to be the most common presentations (60–66% of patients) in adults (27). HE with cerebellar ataxia as the main clinical symptom is quite rare.

### Diagnostic criteria

HE has no definitive diagnostic criteria and depends on the diagnosis of exclusion, due to its low incidence, diverse clinical presentation, and unknown specific pathogenesis (28). Abnormally elevated thyroid antibodies, including anti-TPO or anti-TG, are present in most cases and are necessary for the diagnosis of HE (28, 29). The most common detected antithyroid antibody is anti-TPO. Elevated protein in CSF occurs in most patients and decreases with disease treatment (30). In our case, the SCF protein level was increased, but regretfully, we did not perform detection for CSF antithyroid antibodies. Moreover, the amino (NH2) terminal region of alpha-enolase (NAE) was an antigen identified in HE patients' brain tissue, and NAE antibodies were elevated in the majority of diagnosed HE patients (31). Some scholars have suggested that NAE antibodies appear to be a useful diagnostic marker of HE (31–33). Anti-NAE antibodies together with antithyroid antibodies will improve the sensitivity of HE in the clinical setting. The majority of patients with HE have normal brain MRI findings (34) although abnormal MRI findings may include ischemic lesions, demyelination, edema, and atrophy (35). The most common EEG feature is diffuse slow wave activity, which reflects CNS involvement and also enables monitoring of the effectiveness of drug therapy.

A number of experts proposed a relatively authoritative diagnostic criterion for HE using a clinical approach in Lancet Neurology in 2016. HE can be diagnosed if a patient meets all of the following criteria: (1) encephalopathy with seizures, myoclonus, hallucinations, or stroke-like episodes; (2) subclinical or mild overt thyroid disease (usually hypothyroidism); (3) brain MRI normal or with non-specific abnormalities; (4) presence of thyroid antibodies in the serum (thyroid peroxidase, thyroglobulin); (5) absence of well-characterized neuronal antibodies in the serum and CSF; and (6) reasonable exclusion of alternative causes (36).

Encephalitis patients presenting with behavioral disturbances, delirium, psychosis, hallucinations, and mood alterations, particularly females with a familial history of auto-immune disease, should be strongly suspected to have HE (9, 29, 37). The diagnostic tests for HE include EEG and serum anti-TPO antibody levels. MRI and lumbar puncture are suggested to rule out infection, stoke, and tumor.

### Treatment

Corticosteroids are the first choice for the treatment of HE and have provided complete remission of symptoms in about 50% of patients after post-hormonal therapy (38). Approximately 40% of patients do not recur after the first course of corticosteroid pulse therapy (39, 40). The doses commonly used in clinical practice are oral prednisone (50–150 mg/d or 1–2 mg/kg/d) for patients with mild symptoms and high-dose methylprednisolone (500–1,000 mg/d) intravenous injection (IV) for those in severe condition (17, 41). In patients resistant to corticosteroids, combination therapy with immunosuppressive medications, such as azathioprine, cyclophosphamide, and methotrexate, is suggested (42–44). Relapse of HE even with high-dose methylprednisolone IV in some patients should prompt early intervention with these immunosuppressive drugs (37). In HE patients who present with paraneoplastic opsoclonus syndrome, while primary tumor treatment, adjunct therapy with immunosuppressive medications, such as rituximab and an anti-CD20 monoclonal antibody, has also been proven to be effective (45). Patients who are unable to tolerate taking corticosteroids or immunosuppressants may be treated with plasma exchange and intravenous immunoglobulin (IVIG). Plasma exchange has been shown to remove anti-TPO. However, no clinical or neurophysiologic improvement was observed despite the documented reduction of the anti-TPO antibody to levels below the limits of laboratory detection in HE patients (21). Steroid therapy is a specific treatment, and improvement with corticosteroids may confirm the diagnosis of HE (10). Although the majority of HE patients respond to steroid treatment, it has been suggested that the lack of response to steroid treatment should not exclude the diagnosis of HE (38). If HE is clinically diagnosed, early intervention with steroids should be initiated (46), and treatment does not differ according to the presence or absence of cerebellar ataxia.

### HE with cerebellar ataxia

Cases with cerebellar involvement or ataxia as the main HE symptom are reported relatively rarely and are easily overlooked in clinical practice. Herein we report an unusual case of HE with cerebellar ataxia (henceforth referred to as HECA) as the main clinical manifestation. The patient presented with dizziness, cerebellar dysarthria, and trunk and limb ataxia and met all the above criteria (36) including mildly elevated CSF protein, elevated serum thyroid antibody titers, normal thyroid function, normal cranial MRI, abnormal thyroid color ultrasound indicating Hashimoto's thyroiditis, absence of well-characterized neuronal antibodies in the serum and CSF, and marked improvement of ataxia symptoms after corticosteroid treatment. For a more comprehensive understanding, we searched PubMed by entering the keyword “Hashimoto encephalitis, cerebellar syndrome, ataxia and cerebellar ataxia” and then identified 20 representative cases (not including the present case) for further analysis in this review. Table 1 summarized the epidemiological and clinical characteristics, laboratory features, brain MRI, treatment, and outcomes of all the 20 cases.

**Table 1:** The clinical characteristics of cases of HE with cerebellar ataxia as the main symptom.

**Year/Country**	**Sex/Age**	**Clinical characteristics**			**Anti-TPO/TG**	**CSF**			**Cranial MRI**	**EEG**		**Time from onset to treatment (months)**	**Steroids therapy**		**Outcome**	**REFS**
		**Dysar-thria**	**Ataxia**	**Others**		**WBC**	**Protein(g/L)**	**Glucose**		**Wave-activity**	**Location**		**Initial dose(g/day) × day**	**Maintenance treatment**		
2017/Spain	F/47	+	+	Nystagmus hypotonia	+/-	NORM	0.678	NORM	MCA	slow wave	diffuse background	6.5	1.0 × 5	tapering prednisone until maintaining 10 mg/day × 6 months	IMP	(50)
2015/Korea	M/30	-	+	Nystagmus	-/+	NORM	NORM	NORM	NORM	NORM	-	9	1.0 × 5	prednisolone 60 mg/day initially and gradually reduced to 20 mg/day within 20 days and maintained for 1 month, then 10 mg/day × 9 months	IMP	(47)
2014/China	F/56	+	+	somniloquy, delirim	+/+	NORM	1.056	UNSP	NORM	fast wave; slow wave	a fast θ wave: the central region of the frontal region; slow wave: diffuse background	3	0.5 × 3	Prednisolone: 30 mg/day × 10 days, 25 mg/day × 10 days, 20 mg/day × 10 days, 15 mg/day × 10 days, 10 mg/day × 10 days, 5 mg/day × 30 days	IMP	(23)
2013/Japan	M/52	-	+	Cognitive impairment/psychiatric symptoms	+/+	UNSP	NORM	UNSP	NORM	slow wave	diffuse background	120	UNSP	UNSP	IMP	(49)
2013/Japan	F/46	+	+	Cognitive impairment/psychiatric symptoms	+/+	UNSP	NORM	UNSP	NORM	NORM	-	12	UNSP	UNSP	IMP	(49)
2013/Japan	F/63	-	+	tremor	+/+	UNSP	NORM	UNSP	NORM	UNSP	UNSP	1	UNSP	UNSP	IMP	(49)
2013/Japan	F/66	+	+	tremor	+/+	UNSP	NORM	UNSP	NORM	NORM	-	2	UNSP	UNSP	IMP	(49)
2013/Japan	F/46	-	+	cognitive impairment/psychiatric symptoms, unconsciousness, myoclonus	+/+	UNSP	NORM	UNSP	NORM	slow wave	UNSP	12	-	IVIG and immunosuppressant (dose UNSP)	DTR	(49)
2013/Japan	F/84	+	+	NORM	+/+	UNSP	NORM	UNSP	MCA	NORM	-	72	UNSP	UNSP	DTR	(49)
2013/Japan	M/55	+	+	NORM	+/-	UNSP	NORM	UNSP	MCA	NORM	-	4	UNSP	UNSP	DTR	(49)
2013/Japan	M/55	+	+	cognitive impairment/psychiatric symptoms	+/-	UNSP	↑	UNSP	NORM	slow wave	UNSP	3	UNSP	UNSP	IMP	(49)
2013/Japan	M/54	+	+	NORM	+/+	UNSP	UNSP	UNSP	MCA	UNSP	UNSP	120	UNSP	UNSP	IMP	(49)
2013/Japan	M/61	+	+	nystagmus	+/+	UNSP	NORM	UNSP	NORM	NORM	-	12	UNSP	UNSP	IMP	(49)
2013/Japan	F/57	-	+	NORM	+/+	UNSP	NORM	UNSP	MCA	NORM	-	12	UNSP	UNSP	DTR	(49)
2013/Japan	F/46	-	+	nystagmus, tremor	+/-	UNSP	NORM	UNSP	MCA	NORM	-	6	UNSP	UNSP	DTR	(49)
2011/China	M/39	+	+	right central facial weakness, lingual fasciculations, briskjawjerk, hyperactivegag reflex	+/+	NORM	1.26	NORM	MCA	NORM	-	UNSP	1.0 × 5	tapering prednisone (dose UNSP)	IMP	(51)
2011/India	F/17	-	+	diplopia	+/UNSP	UNSP	0.52	UNSP	NORM	NORM	-	6.5	1.0 × 5	six pulses of steroids (once a month) and oral thyroxine 100 ug/day	IMP	(52)
2011/India	M/47	+	+	NORM	+/UNSP	UNSP	NORM	NORM	NORM	NORM	-	6	1.0 × 5	four pulses of steroids	IMP	(52)
2007/Japan	F/41	+	+	NORM	+/+	UNSP	NORM	UNSP	NORM	slow wave	diffuse background	9	1.0 × 3	oral administration of prednisolone 30 mg/day	IMP	(33)
2002/Athens	F/47	+	+	nystagmus	+/+	UNSP	0.70	UNSP	NORM	slow wave	diffuse background	UNSP	16 mg of prednisolone three times daily followed by a tapering dose	IVIG	IMP	(48)

MCA, mild cerebellar atrophy; NORM, normal; UNSP, unspecified; DTR, deteriorated; IMP, improved; REFS, references.

#### Epidemiology of HECA

According to our statistical analysis, there are ethnic differences in the prevalent population, with 18 patients (90%) of Asian origin and two patients (10%) of Europe origin. The patients consisted of 8 men (40%) and 12 women (60%), and the average patient age was 50 years (range: 17–84). This is consistent with the previous overall data of HE that patients are predominantly female and disease usually occurs in their 5–6th decades of life (12, 13). The different incidence rates in men and women from previous literature (10, 11) may be related to our small sample size.

#### Additional symptoms of HECA

All patients presented symptoms of ataxia, 13 patients (65%) also had dysarthria and 14 patients (70%) had other symptoms (covering hypotonia, nystagmus, diplopia, psychiatric symptoms, lightheadedness, and cognitive/hearing impairment or tremor). Only one female patient (23) claimed that she had ulcerative colitis 3 months before, which is a collection of chronic and recurrent inflammatory illnesses of the gut produced by aberrant immune-mediated diseases of various etiologies. Other cases did not mention the presence of other autoimmune diseases.

#### Laboratory examination of HECA

A total of 13 patients (65%) were positive for both serum anti-TPO and anti-TG antibodies, six patients (30%) showed positive anti-TPO antibodies alone, and one patient (47) was negative for serum anti-TPO antibodies and positive for anti-TG antibodies. In terms of thyroid function, most cases (12 patients, 60%) had normal thyroid function, two patients (10%) showed hyperthyroidism, two patients (10%) showed hypothyroidism, and four patients showed indeterminate thyroid function. The data showed no specific changes in CSF, with most patients (13 patients, 65%) having normal CSF proteins and six patients (30%) having mildly elevated CSF proteins, with a mean value of 0.84 g/L (range: 0.52–1.26 g/L). Intracranial pressure, white blood cell count, and glucose concentration were all in the normal range (except for 15 patients not mentioned in the references). No significant abnormalities were seen on MRI except for mild cerebellar atrophy (seven patients, 35%). A total of 11 patients (55%) had no significant EEG abnormalities, six patients (30%) showed diffuse background slow waves, and 1 case specifically showed a high-power θ wave in the central region of the frontal region and diffuse background slow waves.

#### Treatment and prognosis of HECA

The intervals between symptom onset and treatment start ranged from 1 month to 10 years (mean duration: 2.5 years), indicating the difficulty of disease recognition. Corticosteroid therapy was given to almost all of the patients (19 patients, 95%), and 15 patients experienced gradual relief of symptoms, including ataxia, dysarthria, and tremor, after receiving corticosteroid therapy. The first dose of corticosteroids varied from 0.5 to 1.0 g/day, applied continuously for 3–5 days in the seven patients who received corticosteroid therapy, and in 5 patients of them, it was gradually reduced to oral low-dose prednisone maintenance therapy for a maximum of 9 months. Anti-TPO or anti-TG antibody titers decreased or even returned to normal levels in 4 patients, and EEG improved in 1 patient (1/7,14%) (33). One patient (48) received IVIG of 6 g four times daily for 6 days and levothyroxine sodium (75 ug/day) with a slight improvement in dysarthria and ataxia symptoms. After 3 months, this patient was given IVIG along with methylprednisolone 16 mg three times daily while continuing sodium thyroxine treatment, followed by a tapering dose. This patient's symptoms further improved. One patient (49) was not treated with corticosteroid therapy for severe diabetes mellitus and was given IVIG and immunosuppressive therapy, but only limited recovery was achieved and severe sequelae remained. A total of 4 patients (20%) did not achieve significant effects after steroid therapy, probably due to their initial severe disease or other reasons. In summary, it can be seen that HE is not a self-limited disease, the vast majority of it is effective against steroids or immunoglobulin therapy. Consequently, if HE is definitely diagnosed, steroids or immunoglobulins should be used as soon as possible. There is no uniform dosage and duration of steroid therapy, which can be given as methylprednisolone (500–1,000) mg/d intravenously for 5 days and prednisone (1–2) mg/kg·d orally, and gradually reduced. Most patients improve within 10 days of steroid therapy (25), and the medication can be repeated for those with recurrent symptoms. The prognosis of HE is optimistic if the treatment is reasonable and timely. Only one patient (23) was followed up for more than 1 year and the patient recovered well-with no recurrence.

In total, we summarized the clinical characteristics of 20 cases of HE with cerebellar symptoms as the main manifestation. These patients were predominantly female (60%) with a mean age of 50 years and presented mainly with ataxia of the limbs or trunk and dysarthria. All patients showed elevated anti-TPO and/or anti-TG antibodies, and no specific abnormalities on brain MRI. 30% (six patients) had mildly elevated CSF proteins. Despite the effectiveness of corticosteroids or IVIG treatment, the time from the symptom onset to the initiation of steroid therapy was 23.1 ± 38.5 months in these 20 cases. It is easy to delay treatment due to the long diagnosis period. In conclusion, HE with cerebellar ataxia as the main symptom is not easy to recognize and often misses the best opportunity for treatment. Therefore, it is necessary to pay attention to this rare manifestation of HE in clinical practice and to diagnose and treat it as early as possible.

## Conclusions

Based on our observations, the importance of recognizing HE is that it is treatable, and most patients have an obvious therapeutic response to corticosteroids or other immunosuppressive agents. We reported an uncommon case of HE with cerebellar ataxia as the main manifestation and reviewed the clinical features of 20 cases of HE with cerebellar ataxia as the core symptoms to highlight the rare clinical manifestation of HE in this clinical setting. Our case and most of the HE cases summarized showed elevated serum anti-TPO/TG, normal or reduced thyroid function, abnormal thyroid color ultrasound indicating Hashimoto's thyroiditis, cranial MRI showing only mild cerebellar atrophy or approximately normal, CSF showing mildly elevated protein, normal cell count and sugar and chloride, no well-characterized neuronal antibodies in serum and CSF, good response to steroids, etc., all of which greatly suggested the diagnosis of HE. Hence, HE should be considered in all patients presenting with encephalopathies, particularly especially in females with histories of auto-immune disease, followed by an early examination of serum thyroid antibodies, thyroid ultrasound, CSF, cranial MRI, and EEG to avoid misdiagnosis and omission, and timely treatment with corticosteroids or further immunoglobulins or immunosuppressive agents once the diagnosis is clear. In addition, patients with HE should be followed up for a long time to monitor disease recurrence or progression, so as to provide more adequate evidence for future disease diagnosis and treatment.

## Data availability statement

The original contributions presented in the study are included in the article/supplementary material, further inquiries can be directed to the corresponding author/s.

## Ethics statement

Written informed consent was obtained from the individual(s) for the publication of any potentially identifiable images or data included in this article.

## Author contributions

CW performed case information collection, literature review, literature information statistics, and drafted the manuscript. YS, WZ, and TS contributed to case information collection and literature information statistics. ZW, YW, and ML contributed to the literature review and manuscript preparation. YZ and LS performed a manuscript review and final version approval. All authors contributed to the article and approved the submitted version.

## Funding

This study was supported by the grant provided by the Major Chronic Disease Program of the Ministry of Science and Technology of China (No. 2018YFC1312301).

## Conflict of interest

The authors declare that the research was conducted in the absence of any commercial or financial relationships that could be construed as a potential conflict of interest.

## Publisher's note

All claims expressed in this article are solely those of the authors and do not necessarily represent those of their affiliated organizations, or those of the publisher, the editors and the reviewers. Any product that may be evaluated in this article, or claim that may be made by its manufacturer, is not guaranteed or endorsed by the publisher.
